# flopp: Extremely Fast Long-Read Polyploid Haplotype Phasing by Uniform Tree Partitioning

**DOI:** 10.1089/cmb.2021.0436

**Published:** 2022-02-16

**Authors:** Jim Shaw, Yun William Yu

**Affiliations:** ^1^ Department of Mathematics, University of Toronto, Toronto, Canada.; ^2^ Computer and Mathematical Sciences, University of Toronto at Scarborough, Scarborough, Canada.

**Keywords:** haplotype phasing, long-reads, polyploid, UPEM

## Abstract

Resolving haplotypes in polyploid genomes using phase information from sequencing reads is an important and challenging problem. We introduce two new mathematical formulations of polyploid haplotype phasing: (1) the min-sum max tree partition problem, which is a more flexible graphical metric compared with the standard minimum error correction (MEC) model in the polyploid setting, and (2) the uniform probabilistic error minimization model, which is a probabilistic analogue of the MEC model. We incorporate both formulations into a long-read based polyploid haplotype phasing method called *flopp*. We show that flopp compares favorably with state-of-the-art algorithms—up to 30 times faster with 2 times fewer switch errors on 6 × ploidy simulated data. Further, we show using real nanopore data that flopp can quickly reveal reasonable haplotype structures from the autotetraploid *Solanum tuberosum* (potato).

## 1. INTRODUCTION

As genomic sequencing technologies continue to improve, we are increasingly able to resolve ever finer genomic details. Case in point, traditional genotyping only determines whether a particular allele is present in a genome (Scheben et al., 2017). However, when organisms are *polyploid* (and most eukaryotic organisms are), they have multiple copies of each chromosome. We are then additionally interested in the problem of resolving *haplotypes*, that is, determining the sequence of alleles on each specific chromosome and not just the presence of an allele within the genome. *Phasing* is the procedure of resolving the haplotypes by linking alleles within a chromosome (Browning and Browning, 2011).

We focus on phasing polyploid organisms by using third-generation sequencing data. Many plants have *ploidy* greater than two (i.e., have more than two copies of each chromosome), such as tetraploid potatoes (*Solanum tuberosum*) or hexaploid wheat and cotton. Haplotype phasing has been used to gain insights into evolution (Eriksson et al., 2018), breeding (Qian et al., 2017), and genome-wide association studies (Maldonado et al., 2019), among other applications.

The most common way of determining haplotypes is to use pooled genotyping information from a population to estimate haplotypes (Browning and Browning, 2011). For unrelated individuals, sophisticated statistical methods are used to determine the most likely haplotypes for each individual (Browning and Browning, 2007; Howie et al., 2009; Delaneau et al., 2019) in the population. For related individuals, identity-by-descent information can be used for haplotype phasing (Gao et al., 2009; Motazedi et al., 2018). However, these types of methods do not work on single individuals because they rely on having population data available.

Instead, in this work, we adopt the approach of single individual phasing by sequencing, which is now common in phasing human haplotypes (Choi et al., 2018). We focus on using sequencing information for phasing, which allows us to phase a single individual without population information or prior haplotype knowledge. This is closely related to genome assembly where overlapping reads are stitched together (Nagarajan and Pop, 2013); in our case, nearby heterozygous alleles are stitched together by read information. For the rest of the article, we use the term “phasing” to mean single individual phasing using sequencing information.

### 1.1. Related work

The first method for phasing polyploid genomes was HapCompass (Aguiar and Istrail, 2012), which uses a graphical approach. Popular methods that followed include HapTree (Berger et al., 2014, 2020), H-PoP (Xie et al., 2016), and SDhaP (Das and Vikalo, 2015). HapTree and H-PoP heuristically maximize a likelihood function and an objective function based on the minimum error correction (MEC) model, respectively whereas SDhaP takes a semi-definite programming approach. HapTree-X (Berger et al., 2020) additionally incorporates long-range expression correlations to allow phasing even of pairs of variants that cannot be covered by a single read, overcoming some of the problems with short-read phasing.

Due to the increased prevalence of long-read data from Oxford Nanopore or PacBio, newer methods taking advantage of the longer-range correlations that are accessible through long-read data have been proposed (Schrinner et al., 2020; Abou Saada et al., 2021). Unfortunately, because the error profiles of long-read technologies differ considerably from Illumina short-reads [e.g., a higher prevalence of indel errors compared to single nucleotide polymorphisms (SNPs)]. Methods tailored to short-reads (Siragusa et al., 2019; Moeinzadeh et al., 2020) may be ineffective, so altogether new paradigms are required.

At a more theoretical level, in the diploid setting, the standard MEC model (Bonizzoni et al., 2016) has proven to be quite powerful. It is known to be APX-Hard and NP-Hard but heuristically solved in practice with relatively good results. Unfortunately, a good MEC score may not imply a good phasing when errors are present (Majidian et al., 2020). This shortcoming is further exacerbated in the polyploid setting, because similar haplotypes may be clustered together since the MEC model does not consider coverage; this phenomenon is known as genome collapsing (Schrinner et al., 2020). Thus, although the MEC model can be applied to the polyploid setting, it may be suboptimal; however, there is yet to be an alternative commonly agreed-upon formulation of the polyploid phasing problem. Indeed, this is reflected in the literature: The mathematical underpinnings of the various polyploid phasing algorithms are very diverse.

### 1.2. Contributions

In this article, we first address the theoretical shortcomings highlighted earlier by giving two new mathematical formulations of polyploid phasing. We adopt a probabilistic framework that allows us to (1) give a better notion of haplotype similarity between reads and (2) define a new objective function, the uniform probabilistic error minimization (UPEM) score. Further, we introduce the idea of framing the polyploid phasing problem as one of partitioning a graph to minimize the sum of the max spanning trees within each cluster, which, we show, is related to the MEC formulation in a specific case.

We argue that these formulations are better suited for polyploid haplotype phasing using long-reads. In addition to our theoretical justifications, we also implemented a method we call flopp (fast local polyploid phaser). flopp optimizes the UPEM score and builds up local haplotypes through the graph partitioning procedure described. When tested on simulated datasets, flopp produces much more accurate local haplotype blocks than other methods and also frequently produces the most accurate global phasing. flopp's runtime is additionally comparable to, and often much faster than, its competitors.

The code for flopp is available at https://github.com/bluenote-1577/flopp. flopp utilizes Rust-Bio (Kster, 2016), is written entirely in the rust programming language, and is fully parallelizable. flopp takes as input either binary alignment map + variant call format (BAM + VCF) files, or the same fragment file format used by AltHap (Hashemi et al., 2018) and H-PoP.

## 2. METHODS

### 2.1. Definitions

We represent every read as a sequence of variants (rather than as a string of nucleotides, which is commonly used for mapping/assembly tasks). Let *R* be the set of all reads that align to a chromosome and *m* be the number of variants in our chromosome. Assuming that tetra-allelic SNPs are allowed, every read *r_i_* is considered as an element of the space $r_{i}\in {\left\{-,0,1,2,3\right\}}^{m}$. A read in this variant space is sometimes called a fragment. Denoting $r_{i}\left[j\right]$ as the *j*th coordinate of *r_i_*, $r_{i}\left[j\right]\in \left\{0,1,2,3\right\}$ if the *j*th variant is contained in the read *r_i_* where 0 represents the reference allele, 1 represents the first alternative allele, and so forth. $r_{i}\left[j\right]=-$ if *r_i_* does not contain the *j*th allele.

We note that flopp by default only uses SNP information, but the user may generate their own fragments, permitting indels and other types of variants to be used even if there are more than four possible alleles. The formalism is the same regardless of the types of variants used or the number of alternative alleles.

For any two reads $r_{1},r_{2}$, let

$$d\left(r_{1},r_{2}\right)=|\left\{k:r_{1}\left[k\right]\neq r_{2}\left[k\right],\left(r_{1}\left[k\right]\neq -\right)\wedge \left(r_{2}\left[k\right]\neq -\right)\right\}|$$

$$s\left(r_{1},r_{2}\right)=|\left\{k:r_{1}\left[k\right]=r_{2}\left[k\right],\left(r_{1}\left[k\right]\neq -\right)\wedge \left(r_{2}\left[k\right]\neq -\right)\right\}|.$$

*d* and *s* stand for *different* and *same*, representing the number of different and same variants, respectively, between two reads.

We use *k* to denote the ploidy. Given a *k*-ploid organism, a natural approach to phasing is to partition *R* into *k* subsets where the cluster membership of a read represents which haplotype the read belongs to. Let $R_{1},\ldots ,R_{k}$ be a partition of *R*. Given a partition $P=\left\{R_{1},\ldots ,R_{k}\right\}$, we denote $P\left[i\right]=R_{i}$.

Define the *consensus haplotype* $H\left(R_{i}\right)\in {\left\{-,0,1,2,3\right\}}^{m}$ associated to a subset of reads as follows. For all indices l=1,…,m let $H\left(R_{i}\right)\left[l\right]=arg{max}_{a}|\left\{r\in R_{i}:r\left[l\right]=a\right\}|$ and break ties according to some arbitrary order. If only − appear at position *l* over all reads, we take $H\left(R_{i}\right)\left[l\right]=-$. It is easy to check that $H\left(R_{i}\right)$ is a sequence in ${\left\{-,0,1,2,3\right\}}^{m}$ such that $H\left(R_{i}\right)\left[k\right]\neq -$ at indices for which some read overlaps, and $\sum_{r\in R_{i}}d\left(H\left(R_{i}\right),r\right)$ is minimized.

In our formalism, we can phrase the MEC model of haplotype phasing as the task of finding a partition $\left\{R_{1},\ldots ,R_{k}\right\}$ of *R* such that $\sum_{i=1}^{k}\sum_{r_{j}\in R_{i}}d\left(r_{j},H\left(R_{i}\right)\right)$, which is called the MEC score, is minimized. For notational purposes, for a subset $R_{i}\subset R$, define $S\left(R_{i}\right)=\sum_{r\in R_{i}}s\left(H\left(R_{i}\right),r\right)$ and $D\left(R_{i}\right)=\sum_{r\in R_{i}}d\left(H\left(R_{i}\right),r\right)$. $\sum_{i=1}^{k}D\left(R_{i}\right)$ is just the MEC score for a particular partition.

### 2.2. Problem formulation

#### 2.2.1. min-sum max tree partition model

**FIG. 1.**
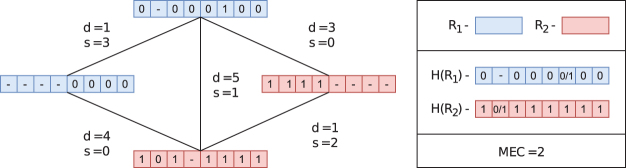
An example of a read-graph (without the weighting *w* specified) along with corresponding d,s values between reads. The colors represent a partition of the read-graph into two subsets $R_{1},R_{2}$. The consensus haplotypes $H\left(R_{1}\right),H\left(R_{2}\right)$ are shown, where the 0∕1 indicates that either 1 or 0 are valid alleles for a consensus haplotype.

Let G(R)=(R,E,w) be an undirected graph where the vertices are *R* and edges *E* are present between two reads $r_{1},r_{2}$ if $r_{1},r_{2}$ overlap, that is, $d\left(r_{1},r_{2}\right)+s\left(r_{1},r_{2}\right)>0$. Let the weight of $e=\left(r_{1},r_{2}\right)$ be $w\left(e\right)=w\left(r_{1},r_{2}\right)$ for some weight function *w*. We call G(R) the *read-graph*; see Figure 1. A similar notion is found (Mazrouee and Wang, 2014, 2020; Das and Vikalo, 2015; Schrinner et al., 2020; Abou Saada et al., 2021).

For a partition of *R* into disjoint subsets $\left\{R_{1},\ldots ,R_{k}\right\}$, we take $G\left(R_{i}\right)$ as defined earlier. We only consider partitions of vertices such that all $G\left(R_{i}\right)$ are connected, which we will denote as valid partitions. Let MST(G) be the maximum spanning tree of a graph *G*. Define

(1) $${SMTP}_{R}^{k}\left(R_{1},\ldots ,R_{k}\right)=\sum_{i=1}^{k}\sum_{e\in MST\left(G\left(R_{i}\right)\right)}w\left(e\right).$$

We formulate the *min-sum max tree partition (MSMTP) problem* as finding a valid partition $\left\{R_{1},\ldots ,R_{k}\right\}$ of *R* such that ${SMTP}_{R}^{k}\left(R_{1},\ldots ,R_{k}\right)$ is minimized. We refer to the *objective function being minimized* as the SMTP score and *the computational problem of minimizing the SMTP score* as the MSMTP problem.

The MSMTP problem falls under a class of problems called graph tree partition problems (Cordone and Maffioli, 2004), most of which are NP-Hard. The following proof shows that MSMTP is NP-Hard.

**Theorem 1.** *MSMTP is NP-Hard for* k≥3.

*Proof.* Let G=(V,E,w) be a connected, undirected, and weighted graph with weight function *w*. We take a reduction from graph coloring.

Let G′=(V,E′,w′) be a complete graph where the vertices of G′ are the same as *G*. Let the weight of any edge e∈E′ to be 2 if e∈E, the original graph, and let it be 1 otherwise.

Let $V_{1},\ldots ,V_{k}$ be a (valid) partition of *V* that solves MSMTP for G′. Note that none of $V_{1},\ldots ,V_{k}$ are empty, otherwise we could move a single vertex to the empty set and it would have a lower SMTP value for this graph. Therefore, the total number of edges over all spanning trees of $G\prime \left(V_{1}\right),\ldots ,G\prime \left(V_{k}\right)$ is $\sum_{i=1}^{k}|V_{i}|-1=|V|-k$. I claim that a *k*-coloring of *G* exists if and only if ${SMTP}_{G\prime }^{k}\left(V_{1},\ldots ,V_{k}\right)=|V|-k$.

If ${SMTP}_{G\prime }^{k}\left(V_{1},\ldots ,V_{k}\right)=|V|-k$, then the maximum spanning tree for each subset only contains edges with weight 1. In particular, this means that no subgraph $G\prime \left(V_{i}\right)$ has an edge with weight 2, so there are no edges between any vertices of *V_i_* in *G*; otherwise, the weight 2 edge would be included in the max spanning tree. Thus, the partition $V_{1},\ldots ,V_{k}$ gives a k−coloring of G.

For the other implication, clearly if a k−coloring exists for *G*, then we can find a partition $V_{1},\ldots ,V_{k}$ such that $G\prime \left(V_{i}\right)$ only has edges of weight 1 between vertices. Then ${SMTP}_{G\prime }^{k}\left(V_{1},\ldots ,V_{k}\right)=|V|-k$ follows.

Therefore, any algorithm that solves MSMTP also decides whether *G* has a *k*-coloring, which is NP-Complete for k≥3.

Intuitively, assuming each $G\left(R_{i}\right)$ is connected, a maximum spanning tree is a maximum measure of discordance along the entire haplotype. We prove next that under a specific constraint on the read-graph, the SMTP score for $w\left(r_{1},r_{2}\right)=d\left(r_{1},r_{2}\right)$ is an upper bound for the MEC score.

**Theorem 2.** *Suppose* $w\left(r_{1},r_{2}\right)=d\left(r_{1},r_{2}\right)$. *Let* a,b∈ℕ *and let R be a set of fragments such that for every* r∈R, *for all* k∈{a,a+1,…,b}, r[k]⁄=− *and for* l⁄∈{a,a+1,⋯,b}, r[l]=−. *For any R_i_ in a valid partition* $\left\{R_{1},\ldots ,R_{k}\right\}$ *of R*,

$$\sum_{e\in MST\left(G\left(R_{i}\right)\right)}w\left(e\right)+\min_{r\in R_{i}}d\left(r,H\left(R_{i}\right)\right)\geq \sum_{r\in R_{i}}d\left(H\left(R_{i}\right),r\right).$$

*Therefore,*

SMTPRk(R1,…,Rk)+∑i=1k minr∈Rid(r,H(Ri))≥∑i=1k∑r∈Rid(H(Ri),r).

*Proof.* Take the augmented graph $G\left(R_{i}\cup \left\{H\left(R_{i}\right)\right\}\right)$. It is clear that $\sum_{r\in R_{i}}d\left(H\left(R_{i}\right),r\right)$ is just $\sum_{e\in Star\left(H\left(R_{i}\right)\right)}w\left(e\right)$, where $Star\left(H\left(R_{i}\right)\right)$ is the star-graph having an internal node $H\left(R_{i}\right)$ and every $r\in R_{i}$ is a leaf node.

Now note that $H\left(R_{i}\right)$ is constructed *precisely* as a sequence in ${\left\{-,0,1,2,3\right\}}^{m}$, which is non − at indices a,a+1,…,b that minimizes the sum $\sum_{e\in Star\left(H\left(R_{i}\right)\right)}w\left(e\right)$. For any $r\in R_{i}$, *r* is also non − at the same indices by assumption, so $\sum_{e\in Star\left(r\right)}w\left(e\right)\geq \sum_{e\in Star\left(H\left(R_{i}\right)\right)}w\left(e\right)$.

Removing the node $H\left(R_{i}\right)$ from Star(r) and the corresponding edge, we get a spanning tree of $G\left(R_{i}\right)$; we call this new graph Star(r)′. Thus, $\sum_{e\in Star\left(r\right)\prime }w\left(e\right)+d\left(r,H\left(R_{i}\right)\right)\geq \sum_{r\in R_{i}}d\left(H\left(R_{i}\right),r\right)$, and clearly $\sum_{e\in MST\left(G\right)}w\left(e\right)\geq \sum_{e\in Star\left(r\right)\prime }w\left(e\right)$. The inequality holds for any *r*, so we can choose *r* to minimize $w\left(r,H\left(R_{i}\right)\right)$, completing the proof.

The theorem just cited relies on the assumption that all reads in the set overlap exactly. Although this is obviously not true for the entire genome, flopp takes a local clustering approach where the entire read set *R* is partitioned into smaller local read sets that overlap significantly.

We verified experimentally that the SMTP score for partitions generated by our method has a strong linear dependence on the MEC score when w=d; see Section 3.3. These results justify that minimizing the SMTP score is worthwhile for this specific case. However, we do not have to necessarily use w=d. In Section 2.3, we opt for a more theoretically sound probabilistic weighting.

#### 2.2.2. UPEM model

The SMTP score has problems with collapsing genomes in the same manner the MEC score does; it does not take into account the assumption that coverage should be uniform between haplotypes. Concretely, if a polyploid organism has two identical haplotypes, the reads from both haplotypes may be clustered together in the MEC model and a noisy read may instead be placed in its own cluster.

Let δ represent the probability that a variant is called incorrectly. Let σ∈ℛ be a normalizing constant, and $X_{i}∼Binomial\left(\left\lceil \left(D\left(R_{i}\right)+S\left(R_{i}\right)\right)∕\sigma \right\rceil ,\delta \right)$ be a binomial random variable. Then,

UPEMR(R1,…,Rk)=∑i=1k logPrXi>D(Ri)σ+log[χ2(|R1|,…,|Rk|)].

The $\chi^{2}\left(x_{1},\ldots ,x_{n}\right)$ term is the *p*-value for the $\chi^{2}$ test, whereas the binomial term is a sum of log one-sided binomial tests where the null hypothesis is that the error rate of a clustering is δ. Therefore, the UPEM score is just a sum of log *p*-values.

The UPEM score is a probabilistic version of the MEC model under the hypothesis that the errors and coverage are uniform across haplotypes. The parameter σ is a normalizing constant and is important because if a specific genome has a high rate of heterozygosity and δ is slightly underestimated, then the sample size $D\left(R_{i}\right)+S\left(R_{i}\right)$ is large. The *p*-value associated with the binomial random variable will be extremely small and drown out the contributions from the $\chi^{2}$ term, so σ is a learned dataset specific constant used to keep the two terms balanced.

The UPEM maximization enforces a relatively uniform partition. Further, errors will be distributed among partitions equally due to the non-linearity of the UPEM score; if one cluster is extremely erroneous, the sum of the binomial terms may be higher for a more spread out error even if the overall MEC score is slightly higher. If error and coverage uniformity assumptions are satisfied, clearly these two properties are desirable in an objective function.

### 2.3. Local graph clustering

**FIG. 2.**
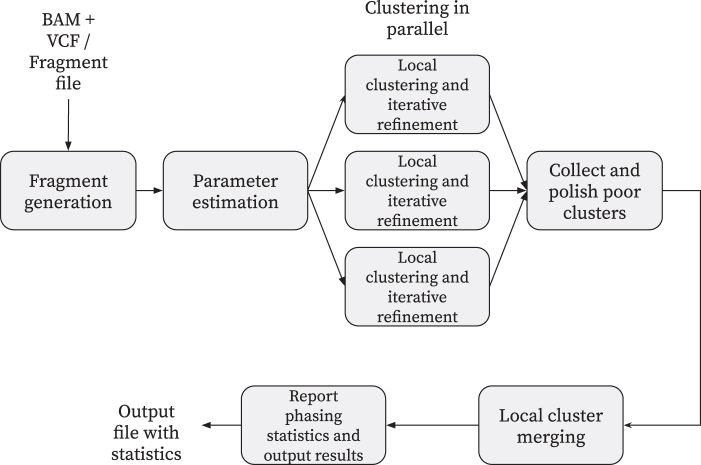
A block diagram outlining flopp's major processing steps. flopp takes BAM+VCF files or a fragment file as input. The fragments are clustered into local haplotype blocks, which are then polished and merged. The final phasing is output to the user as well as various phasing statistics. BAM, binary alignment map; VCF, variant call format.

**FIG. 3.**
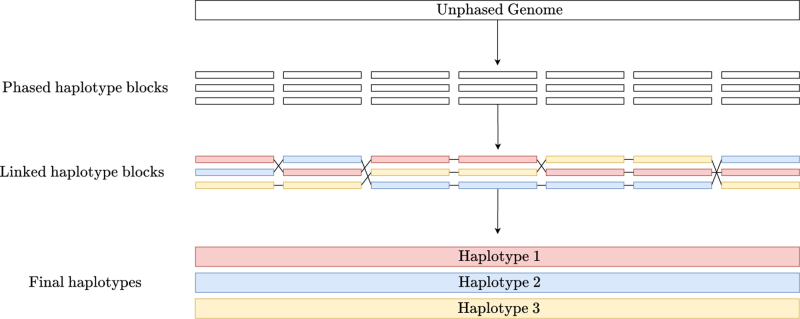
A visual example of the local clustering and linking procedure. flopp breaks up the unphased genome into chunks. The associated reads to each chunk are locally phased and then linked.

We now discuss the algorithms implemented in flopp. A high-level block diagram showing outlining flopp's main processing stages is outlined in Figure 2. Importantly, flopp is a local clustering method, which means that we first attempt to find good partitions for smaller subsets of reads by optimizing the SMTP and UPEM functions and then joining haplotype blocks together afterward. A graphic outlining the local clustering and linking procedure can be found in Figure 3.

#### 2.3.1. Choice of edge weight function

Previous methods that use a read-graph formalism such as SDhaP (Das and Vikalo, 2015), FastHap (Mazrouee and Wang, 2014), WhatsHap Polyphase (WHP) (Schrinner et al., 2020), and nPhase (Abou Saada et al., 2021) all define different weightings between reads. Two previously used weight functions are

$$w_{SDhaP}\left(r_{1},r_{2}\right)=\frac{s\left(r_{1},r_{2}\right)-d\left(r_{1},r_{2}\right)}{s\left(r_{1},r_{2}\right)+d\left(r_{1},r_{2}\right)}\left(SDhaP\right)$$

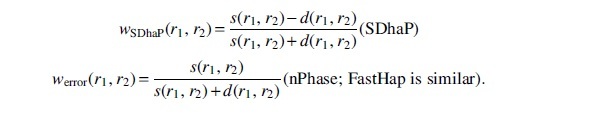


These weightings are quite simple and have issues when the lengths of the reads have high variance, as is the case with long-reads. A more sophisticated approach is to use a probabilistic model. Let $E\left(r_{1},r_{2}\right)=1-w_{error}\left(r_{1},r_{2}\right)$ be the average error rate between two reads. Assuming an error parameter δ as described in the UPEM formula, we define

$$D_{KL}\left(r_{1},r_{2}||\delta \right)=E\left(r_{1},r_{2}\right)\log\left(\frac{E\left(r_{1},r_{2}\right)}{2\delta \left(1-\delta \right)}\right)+\left(1-E\left(r_{1},r_{2}\right)\right)\log\left(\frac{1-E\left(r_{1},r_{2}\right)}{1-2\delta \left(1-\delta \right)}\right).$$

$D_{KL}\left(r_{1},r_{2}||\delta \right)$ is the Kullback-Leibler divergence between a $E\left(r_{1},r_{2}\right)-$coin and a 2δ(1−δ)−coin. The reason we use 2δ(1−δ) is because, given two reads with error rate δ, the probability that two variants from the same haplotype are different is 2δ(1−δ). Now let

(2) $$w\left(r_{1},r_{2}\right)=\left[s\left(r_{1},r_{2}\right)+d\left(r_{1},r_{2}\right)\right]\cdot D_{KL}\left(r_{1},r_{2}||\delta \right).$$

$D_{KL}\left(r_{1},r_{2}||\delta \right)$ is a measure of divergence between the error profiles of the two reads and the expected error profile for two reads from the same haplotype. There is a slight issue that $D_{KL}\left(r_{1},r_{2}||E\right)$ has a minimum at $E\left(r_{1},r_{2}\right)=2\delta \left(1-\delta \right)$ when δ is fixed. We want $D_{KL}$ to be a monotonically increasing function with respect to *E*, so we flip the sign of $D_{KL}\left(r_{1},r_{2}||\delta \right)$ if E<2δ(1−δ).

There is a nice interpretation of $w\left(r_{1},r_{2}\right)$ when $E\left(r_{1},r_{2}\right)>2\delta \left(1-\delta \right)$ as given by the following theorem.

**Theorem 3** (Arratia and Gordon (1989)). *Suppose* p<a<1*. Let* $H=a\log\frac{a}{p}+\left(1-a\right)\log\left(\frac{1-a}{1-p}\right)$ *be the Kullback-Leibler divergence between an* a−*coin and a* p−*coin. Then*

(3) $$\mathrm{Pr}\left(Binom\left(n,p\right)\geq an\right)\leq e^{-nH}.$$

*In particular, this bound is asymptotically tight up to logarithms,* that is,

(4) $$\lim_{n\to \infty }\frac{\log\mathrm{Pr}\left(Binom\left(n,p\right)\geq an\right)}{-nH}=1.$$

The proof of the theorem cited earlier is simple; it follows by an application of a Chernoff bound and Sterling's inequality. Arratia and Gordon (1989) show that this approximation is particularly good when a>>p. Theorem 3 gives an interpretation of $w\left(r_{1},r_{2}\right)$ as the negative log value of a 1-sided binomial test where the null hypothesis is that $r_{1},r_{2}$ come from the same haplotype, the number of trials is $s\left(r_{1},r_{2}\right)+d\left(r_{1},r_{2}\right)$, and the number of successes is $d\left(r_{1},r_{2}\right)$.

WHP (Schrinner et al., 2020) uses a log ratio of binomial probability densities as their edge weight. Mathematically, the resulting formula is almost the same as Equation 2, except they fix $E\left(r_{1},r_{2}\right)$ to be the average error rate between reads from different haplotypes, a constant. They end up with both negative and positive edges with a large magnitude and Theorem 3 does not apply to their case. On the other hand, our edge weights are rigorously justified as negative log *p*-values for a binomial test, making it more interpretable.

#### 2.3.2. MSMTP algorithm

We devised a heuristic algorithm for local clustering inspired by the MSMTP problem. From now on, let S⊂R. The pseudocode is shown in Algorithm 1. In the diploid setting, the main idea behind this algorithm is similar to that of FastHap (Mazrouee and Wang, 2014), but the algorithm is quite different after generalizing to higher ploidies.

For the FindMaxClique method mentioned in Algorithm 1, we use a greedy clique-finding method that takes $O\left(k|S|\log|S|+k^{2}|S|\right)$ time. First, we take the heaviest edge between two vertices and make this our 2-clique. Then, we re-sort vertices by maximizing the minimum distance from the vertex to all vertices in the 2-clique, add a third edge to get a 3-clique, and so forth until we get a k−clique.

**Table:** 

**Algorithm 1:** Greedy min-max read partitioning
**Input:** Read-graph G(S), ploidy *k*, iterations *n*
**Output:** A partition $\left\{S_{1},\ldots ,S_{k}\right\}$ of *S*
**1**$\left\{v_{1},\ldots ,v_{k}\right\}\leftarrow$FindMaxClique(G(S),k)
**2 for** i=1 *to k* **do**
**3** $|S_{i}\leftarrow \left\{v_{i}\right\}$
**4 end**
**5 for** i=1 *to n* **do**
**6** $|V\leftarrow G\left(S\right)\setminus {⋃}_{i=1}^{k}S_{i}$
**7** | Reverse-sort *V* by assigning to v∈V the value
| $v\to \min_{S_{i}\in \left\{S_{1},\ldots ,S_{k}\right\}}\max_{r\in S_{i}}s\left(r,v\right)+d\left(r,v\right)$
**8** |$V\leftarrow V\left[:\left\lceil \frac{\left|V\right|}{n}\right\rceil \right]$
**9** |**for***v in V***do**
**10** |S′←argminSi maxr∈Siw(v,r)
**11** S′←S′∪{v}
**12 end**
**13 end**
**14** Return $\left\{S_{1},\ldots ,S_{k}\right\}$

The complexity of the local clustering algorithm is $O\left(n|S{|}^{2}+k|S|\log|S|+k^{2}|S|\right)$. In practice, note that |S|>>k. The parameter *n* is fixed to be 10. By iterating over *n*, we re-sort the edges based on their overlaps to the new clusters, which have changed since the previous iteration. This improves the order in which we add vertices to the clusters.

The connection to MSMTP is at line 10. A priori, it is not obvious what metric to use to determine which cluster to put the vertex in. For Kruskal's algorithm, one starts by considering the heaviest edges, so we decided to minimize the maximum edge from the vertex to the cluster so that the heaviest edges are small. Intuitively, a maximum spanning tree is sensitive to a highly erroneous edge, so we prioritize minimizing the worst edge even if on average the vertex connects to the cluster well.

#### 2.3.3. Iterative refinement of local clusters

We refine the output of the local clustering procedure by optimizing the UPEM score using a method similar to the Kernighan-Lin algorithm (Kernighan and Lin, 1970). Pseudocode can be found in Algorithm 2.

In lines 4–14, we check how swapping vertices between partitions affects the overall UPEM score and take a fraction of the best swaps in line 14. We then execute the swaps and check whether the UPEM score has increased. If it has not, we terminate the algorithm; otherwise, we continue until *n* iterations have passed. We take n=10 in practice, and note that almost always the algorithm terminates before 10 iterations pass. In practice, we set the parameter n=10. The time complexity of Algorithm 2 is O(n|S|klog(|S|k)).

**Table:** 

**Algorithm 2:** Iterative refinement of UPEM score
**Input :** Partition $P=\left\{S_{1},\ldots ,S_{k}\right\}$, iterations *n*
**Output:** A modified optimized partition $P=\left\{S\prime_{1},\ldots ,S\prime_{k}\right\}$
**1 for** *i = 1 to n* **do**
**2** OldScore←UPEM(P)
**3** L←⊘
**4 for** *S_i_ in P* **do**
**5 for** *r in S_i_* **do**
**6 for***S_j_ in P*, $S_{j}\neq S_{i}$**do**
**7 ** Compute change in *UPEM*, $\Delta \left(r,S_{j}\right)$ by moving *r* from
*S_i_* to *S_j_*
**8 ** if $\Delta \left(r,S_{j}\right)>0$**then**
**9 ** $L\leftarrow L\cup \left\{\left(r,S_{i},S_{j},\Delta \left(r,S_{j}\right)\right)\right\}$.
**10 end**
**11 end**
**12 end**
**13 ** **end**
**14 ** L← reverse sort *L* by $L\left[j\right]\to \Delta \left(r,S_{j}\right)$
**15 for**k=1*to*$\left\lceil \frac{\left|S\right|}{n}\right\rceil$) **do**
**16 ** $\left(r,S_{i},S_{j},\Delta \left(r,S_{j}\right)\right)=L\left[k\right]$
**17 ** $S_{j}\leftarrow S_{j}\cup \left\{r\right\}$
**18 ** $S_{i}\leftarrow S_{i}\setminus \left\{r\right\}$
**19 end**
**20 ** NewScore←UPEM(P)
**21 if** NewScore<OldScore **then**
**22 ** | Reverse the moves made in Lines 16-20
**23 ** Return *P*
**24 end**
**25 end**
**26** Return *P*

#### 2.3.4. Local phasing procedure

Note that Algorithms 1 and 2 work on subsets of reads or subgraphs of the underlying read-graph. Let b∈ℕ be a constant representing the length of a local block. We consider subsets $B_{1},\ldots ,B_{l}\subset R$ where

$$B_{i}=\left\{r\in R:\exists j,b\cdot \left(i-1\right)\leq j\leq b\cdot i,r\left[j\right]⁄=-\right\}.$$

The subsets are just all reads that overlap a shifted interval of size *b*, similar to the work done in Sankararaman et al. (2020). After choosing a suitable *b*, we run the read-partitioning and iterative refinement on all $B_{1},\ldots ,B_{l}$ to generate a set of partitions $P_{1},\ldots ,P_{l}$. We found that a suitable value of *b* is the $\frac{1}{3}-$quantile value of read lengths. By read length, we mean the last non ′−′ position minus the first non ′−′ position of r∈{0,1,2,3,−}.

It is important to note that computationally, the local clustering procedure is easily parallelizable. The local clustering step has, therefore, a linear speedup in the number of threads.

### 2.4. Polishing, merging, and parameter estimation

#### 2.4.1. Filling in erroneous blocks

Once we obtain a set of partitions $P_{1},\ldots ,P_{l}$ of $B_{1},\ldots ,B_{l}$ according to the local clustering procedure cited earlier, we can identify partitions with low UPEM score and correct them. After computing the UPEM scores for every partition, we use a simple 3.0 inter-quartile range outlier detection method for the distribution of UPEM scores. For an outlying partition *P_i_* of the read set *B_i_*, if a partition $P_{i-1}$ is not an outlier, we remove *P_i_* and extend $P_{i-1}$ to include *B_i_*. To do this, we run a subroutine of Algorithm 1 where we skip the clique finding procedure and instead treat the partition $P_{i-1}$ as the initial clusters. We then run lines 9–12 of Algorithm 1 where in line 9 we iterate over $v\in B_{i}\setminus B_{i-1}$ instead.

For genomes with large variations in coverage, we give the user the option to disable this procedure because variation in coverage may affect the UPEM score distribution.

#### 2.4.2. Local cluster merging

Let *P* represent the final partition of all reads *R*. We build *P* given $P_{1},\ldots ,P_{l}$ as follows. Start with $P=P_{1}$. Let *S_k_* be the symmetric group on *k* elements, that is, the set of all permutations on k−elements. At the *i*th step of the procedure, let

$$\sigma_{i}={arg max}_{\sigma \in S_{k}}\sum_{j=1}^{k}|P\left[j\right]\cap P_{i+1}\left[\sigma \left(j\right)\right]|.$$

Then, let $P\left[j\right]=P\left[j\right]\cup P_{i+1}\left[\sigma_{i}\left(j\right)\right]$ for all j=1,…,k. Repeat this procedure for i=1,…,l−1.

We experimented with more sophisticated merging procedures such as a beam-search approach but did not observe a significantly better phasing on any of our datasets.

#### 2.4.3. Phasing output

Once a final partition *P* has been found, we build the final phased haplotypes. The output is *k* sequences in ${\left\{0,1,2,3,-\right\}}^{m}$ where *m* is the number of variants. flopp can take fragment files as input, in which each line of the file describes a single read fragment in ${\left\{0,1,2,3,-\right\}}^{m}$, or it can take a VCF file and a BAM file of aligned reads. In the former case, without a VCF file, we do not have genotyping information, so we simply output $\left\{H_{1},\ldots ,H_{k}\right\}$ where $H_{i}=H\left(P\left[i\right]\right)$ is the consensus haplotype. If a VCF file is present, flopp constrains the final haplotype by the genotypes, that is, for some output variant, the number of reference and alternate alleles is the same as in the VCF file.

We constrain the haplotypes using the VCF as follows. For every variant indexed over 1≤i≤m, let c(i,j,a)∈ℛ be a value representing the confidence of calling allele *a* at index *i* for the haplotype represented by P[j]. We produce k− haplotypes according to Algorithm 3.

**Table:** 

**Algorithm 3:** Polishing output haplotypes using genotype information.
**Input:** Partition *P*, Genotyping information
**Output:**k=|P| haplotypes $H_{1},\ldots ,H_{k}\in {\left\{0,1,2,3,-\right\}}^{m}$
**1** Initialize $H_{1},\ldots ,H_{k}$, $H_{i}\left[n\right]=-$ for all 1≤n≤m
** 2 for** i=1 *to m* **do**
** 3 for** j=1 *to k* **do**
** 4 for** a∈{0,1,2,3} **do**
** 5** L←c(i,j,a)
** 6 end**
** 7 end**
** 8** L← reverse-sort *L*
** 9 for** c(i,j,a)∈L **do**
**10 if**$|\left\{H_{n}:H_{n}\left[i\right]=a\right\}|<$ # *of a in VCF file and* $H_{j}\left[i\right]=-$ **then**
**11** $H_{j}\left[i\right]=a$
**12 end**
**13 end**
**14 end**

For the function c(i,j,a) describing the confidence for calling allele *a* at position *m* for haplotype *j*, we choose the function

$$c\left(i,j,a\right)=\frac{\left|\left\{r\in P\left[j\right]:r\left[i\right]=a\right\}\right|}{|\left\{r\in P\left[j\right]:r\left[i\right]\neq a\right\}|+1}.$$

#### 2.4.4. Parameter estimation

We already mentioned in previous sections how we set all parameters for algorithms except for δ and the parameter σ in the UPEM score. We set σ to be the median length of the reads divided by 25, which we empirically found to be a good balance between the binomial and chi-squared terms.

To estimate δ, we start with an initial guess of δ=0.03. We then select 10 subsets $B_{i}\subset R$ at random and perform local clustering and refinement. We estimate δ from the 10⋅k total clusters by choosing δ= the $\frac{1}{10}$th quantile error, where for each cluster $C\subset B_{i}$ the error is $\frac{D\left(C\right)}{S\left(C\right)+D\left(C\right)}$. We pick a bottom quantile because we assume that there is some error in our method, so to get the true error rate we must underestimate the computed errors.

## 3. RESULTS AND DISCUSSION

### 3.1. Phasing metrics

There are a plethora of phasing metrics developed for diploid and polyploid phasing (Motazedi et al., 2017). We use three different metrics of accuracy, but argue that each individual metric can be misleading and that all three metrics should be used in unison before drawing conclusions on phasing accuracy.

For a global measure of goodness of phasing, we use the Hamming error rate. Given a set of true haplotypes H={H[1],…,H[k]} and a set of candidate haplotypes $H^{*}=\left\{H^{*}\left[1\right],\ldots ,H^{*}\left[k\right]\right\}$, we define the Hamming error rate as

$$HE\left(H,H^{*}\right)=\min_{\sigma \in S_{k}}\sum_{i=1}^{k}d\prime \left(H\left[i\right],H^{*}\left[\sigma \left(i\right)\right]\right)∕mk$$

where *S_k_* is the set of permutations on *k* elements, *m* is the length of each H[i], *k* is the ploidy, and d′ is the same as the *d* function defined earlier except that we count the case where one haplotype has a ′−′ at a coordinate as an error.

We define the switch error rate (SWER) similarly to WHP (Schrinner et al., 2020). Let $\Pi_{i}\subset S_{k}$ be the set of permutations such that $H\left[j\right]\left[i\right]=H^{*}\left[j\right]\left[\sigma \left(i\right)\right]$ for all 1≤j≤k. These are the mappings from the truth to the candidate haplotypes that preserve the alleles at position *i*. Then, we define the switch error as

$$SWER\left(H,H^{*}\right)=\min_{\sigma_{1}\in \Pi_{1},\ldots ,\sigma_{n-1}\in \Pi_{n-1}}\frac{1}{n}\sum_{i=1}^{n-1}1_{\sigma_{i}\neq \sigma_{i+1}}$$

where $1_{\sigma_{i}\neq \sigma_{i+1}}=1$ if $\sigma_{i}\neq \sigma_{i+1}$ and 0 otherwise.

The Hamming error, thoughe easily interpretable, can be unstable. A single switch error can drastically alter the Hamming error rate. The SWER also has issues; for example, if two switches occur consecutively, the phasing is still relatively good but the SWER is worse than if only one switch occurred.

We define a new error rate, called the q-block error rate. For a haplotype H[i], break H[i] into non-overlapping substrings of length *q*. Denote each new block 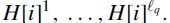. For a set of haplotypes *H*, doing this for every haplotype gives a collection of haplotype blocks $H^{1},\ldots ,H^{\ell_{q}}$. Then, the q-block error rate is


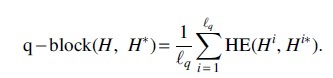


The q-block error rate measures local goodness of assembly and interpolates between the Hamming error rate, when *q* is the size of the genome, and a metric similar to the switch error when q=2.

### 3.2. Simulation procedure

We used the v4.04 assembly of *S. tuberosum* produced by the Potato Genome Sequencing Consortium (Hardigan et al., 2016) as a reference. We took the first 3.5 Mb of chromosome 1 and removed all “N”s, leaving about 3.02 Mb and simulated bi-allelic SNPs by using the haplogenerator software (Motazedi et al., 2017), a script made specifically for realistic simulation of variants in polyploid genomes.

We generated SNPs with a mean distance of 45 and 90 bp between SNPs. This is in line with the 42.5 bp average distance between variants, as seen in Uitdewilligen et al. (2013) for *S. tuberosum*; in that study, they observe that >80% of variants are bi-allelic SNPs. This is also in line with the 58 bp mean distance between variants seen for the hexaploid sweet potato (Ipomoea batatas) observed in Yang et al. (2017b). The dosage probabilities provided to haplogenerators are the same parameters as used in Motazedi et al. (2017) for the tetraploid case. When simulating triploid genomes, we use the same dosage probabilities but disallow the case for three alternate alleles.

We used two different software packages for simulating reads. We used PaSS (Zhang et al., 2019) with the provided default error profiles for simulating PacBio RS reads. PaSS has higher error rates than other methods such as PBSIM (Ono et al., 2013), which tends to underestimate error (Zhang et al., 2019). We used NanoSim (Yang et al., 2017a) for simulating nanopore reads by using a default pre-trained error model based on a real human dataset provided by the software.

After generating the haplotypes and simulating the reads from the haplotypes, we obtain a truth VCF from the haplotypes. We map the reads by using minimap2 (Li, 2018) to the reference genome. The scripts for the simulation pipeline can be found at https://github.com/bluenote-1577/flopp_test.

### 3.3. SMTP versus MEC correlation

We ran flopp on four different simulated datasets and calculated the SMTP score with the weight function $w\left(r_{1},r_{2}\right)=d\left(r_{1},r_{2}\right)$; see Equation 1, and the MEC score for each local partition before merging.

**FIG. 4.**
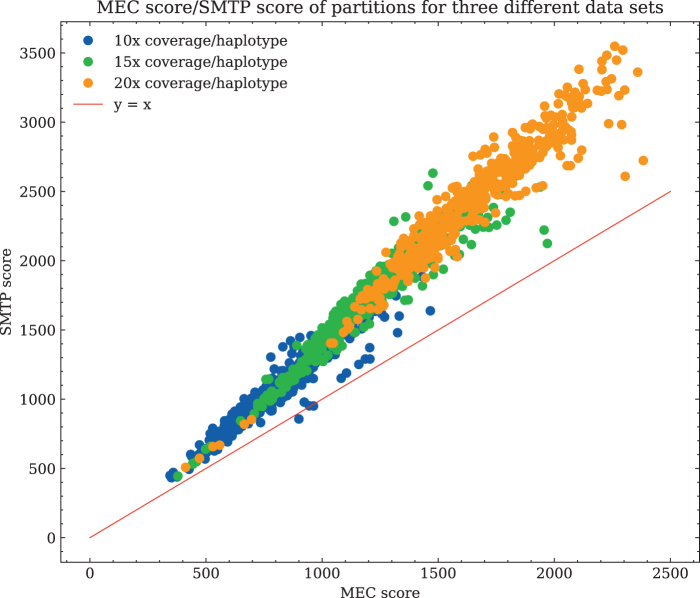
A plot of the SMTP versus MEC score for every local partition (i.e., partition blocks before merging/linking) generated by flopp over three different coverages on 4 × ploidy simulated data, as described in Section 3.2. There is a strong linear relationship between MEC and SMTP scores, and the upper bound for Theorem 2 holds surprisingly well as seen by almost all points being above the line y=x. SMTP, sum max tree partition; MEC, minimum error correction.

We varied the coverage between 10 × to 20 × for a simulated 4 × ploidy genome. We also varied the length of the local partition blocks by manually changing the parameter *b* mentioned at the end of Section 2.3 over three different values (20, 50, and 80 SNPs) for each different dataset to investigate how the size of the local clusters affects the SMTP and MEC relationship. The results are shown in Figure 4.

### 3.4. Results on simulated data set

We primarily test against H-PoPG (Xie et al., 2016), the genotype constrained version of H-PoP. Other methods such as HapTree and AltHap were tested, but we ran into issues with either computing time or poor accuracy due to the methods not being suited for long-read data. We did not test against nPhase, because the output of nPhase does not have a fixed number of haplotypes. We discuss WHP (Schrinner et al., 2020) at the end of this section.

**FIG. 5.**
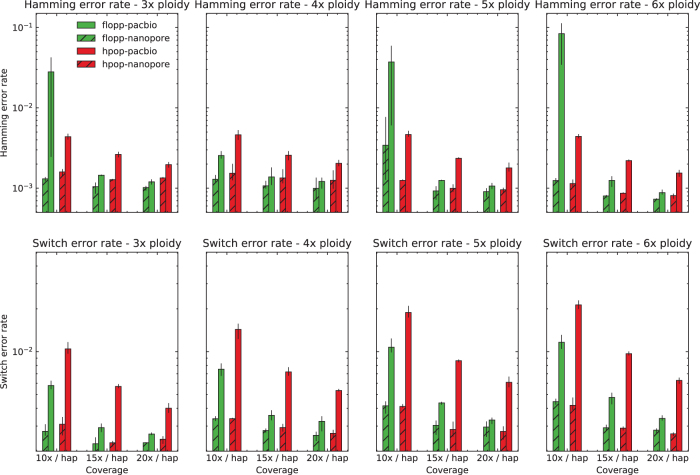
The mean SWER and Hamming error rate from testing on simulated data sets as described in Section 3.2 over a range of ploidies and coverages on two different types of read simulators, with SNPs 45 bp apart on average. The error bars represent the lowest and highest values for the metric over three iterations. The results on the nanopore simulated reads from NanoSim (Yang et al., 2017a) are similar for both methods. flopp achieves much better SWERs on the PacBio simulated reads from PaSS (Zhang et al., 2019), although for low coverage flopp sometimes has a higher Hamming error rate. SNPs, single nucleotide polymorphisms; SWER, switch error rate.

**FIG. 6.**
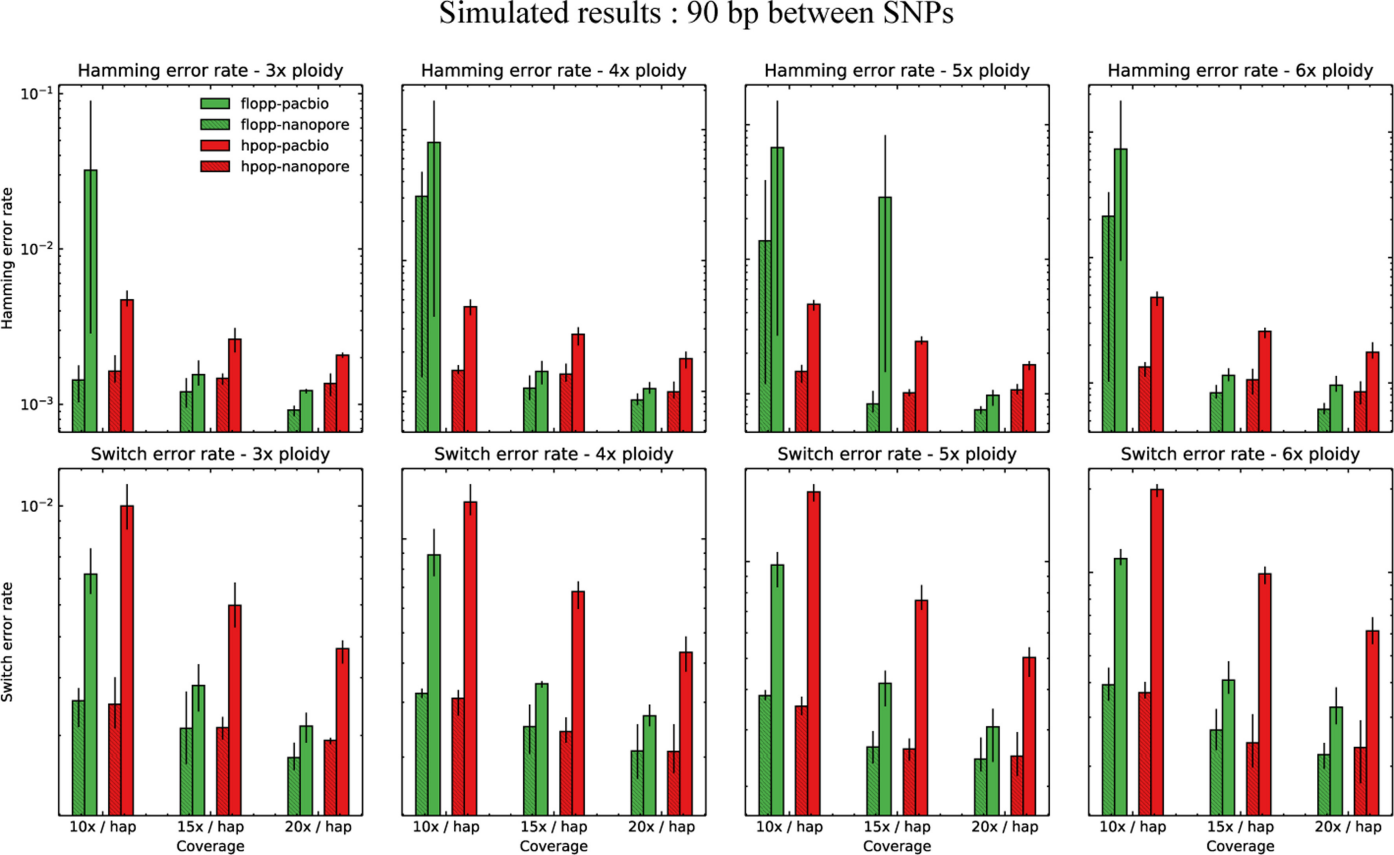
The mean SWER and Hamming error rate from testing on simulated datasets as described in Section 3.2. The same experiment as shown in Figure 5 except 90 bp between SNPs on average.

The switch error and Hamming error rates are shown in Figure 5 for 45 bp average distance between SNPs. For 90 bp, the results are shown in Figure 6. The rest of the analysis in the section pertains to the 45 bp distance case.

**FIG. 7.**
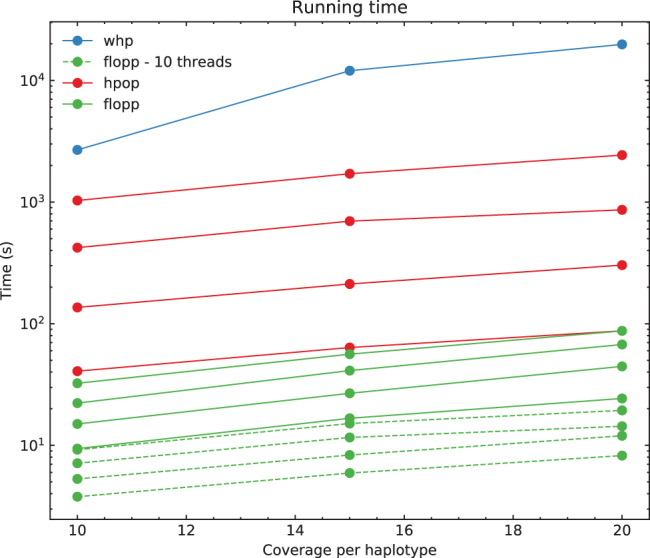
Run times for flopp, H-PoPG, and one instance of WHP. Each line represents a different ploidy, with higher ploidies taking longer. The one instance of WHP was run at 3 × ploidy. H-PoPG; WHP, WhatsHap polyphase.

For each test, we ran the entire pipeline three times; each run at high ploidies takes on the timescale of days to complete. The run times on PacBio reads for H-PoPG, flopp, as well as one instance of WHP on 3 × ploidy data are shown in Figure 7.

For the nanopore dataset simulated from NanoSim, the results for H-PoPG and flopp are very similar. The PaSS PacBio simulator outputs reads that are more erroneous, and we can see that flopp generally performs better than H-PoPG across ploidies except for the Hamming error rate when the coverage is relatively low; interestingly, the SWER is still lower in this case. flopp's SWER is consistently 1.5–2 times lower than H-PoPG for the simulated PacBio reads. On the low coverage datasets, H-PoPG's global optimization strategy leads to a better global phasing than flopp's local methodology.

**FIG. 8.**
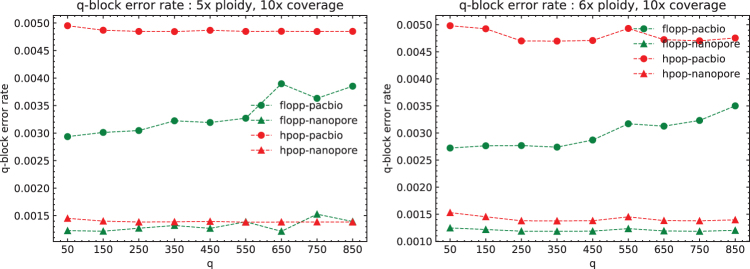
The mean q-block error rates for the 5 × and 6 × ploidy datasets at 10 × coverage per haplotype over three iterations. Each variant is spaced on average 45 bp apart, so each block is of size ∼q⋅45. The upward trend indicates an inaccurate global phasing, but the q-block error rates for flopp are lower than for H-PoP in this regime. H-PoP.

Note that in these tests we phase a contig of length 3.02 Mb, whereas most genes of interest are smaller. In Figure 8, we plot the mean q-block error rates of the 5 × and 6 × ploidy phasings at 10 × coverage; these runs have higher Hamming error rates for flopp than H-PoPG. For blocks of up to around 850⋅45=38250 bases, flopp outputs phasings with lower q-block error rates than H-PoPG despite a larger global error rate. Although flopp may sometimes give worse global phasings than H-PoPG, flopp can give extremely accurate local phasings.

Computationally, Figure 7 shows that flopp is at least 3 times faster than H-PoPG and up to 30 times faster than H-PoPG for 6 × ploidy on a *single thread*. The local clustering step sees a linear speedup from multi-threading. For most datasets, we get a 3–4 times speedup with 10 threads.

We tried testing against WHP (Schrinner et al., 2020), but we found that the accuracy was relatively poor across our datasets and it took a long time to run; see Figure 7. Using the default block-cut sensitivity value, the N50 did not exceed 20 for the 67,000 variants on the simulated contig. We found that WHP takes a conservative approach as >15% of the variants were not called by WHP, contributing to a high hamming error rate. However, we noticed that on real data (discussed below), the runtime of WHP was much more reasonable at 11,492 seconds with 10 threads despite a much larger dataset, suggesting that on certain datasets the performance may be more reasonable.

### 3.5. Phasing tetraploid potato using real nanopore data

**FIG. 9.**
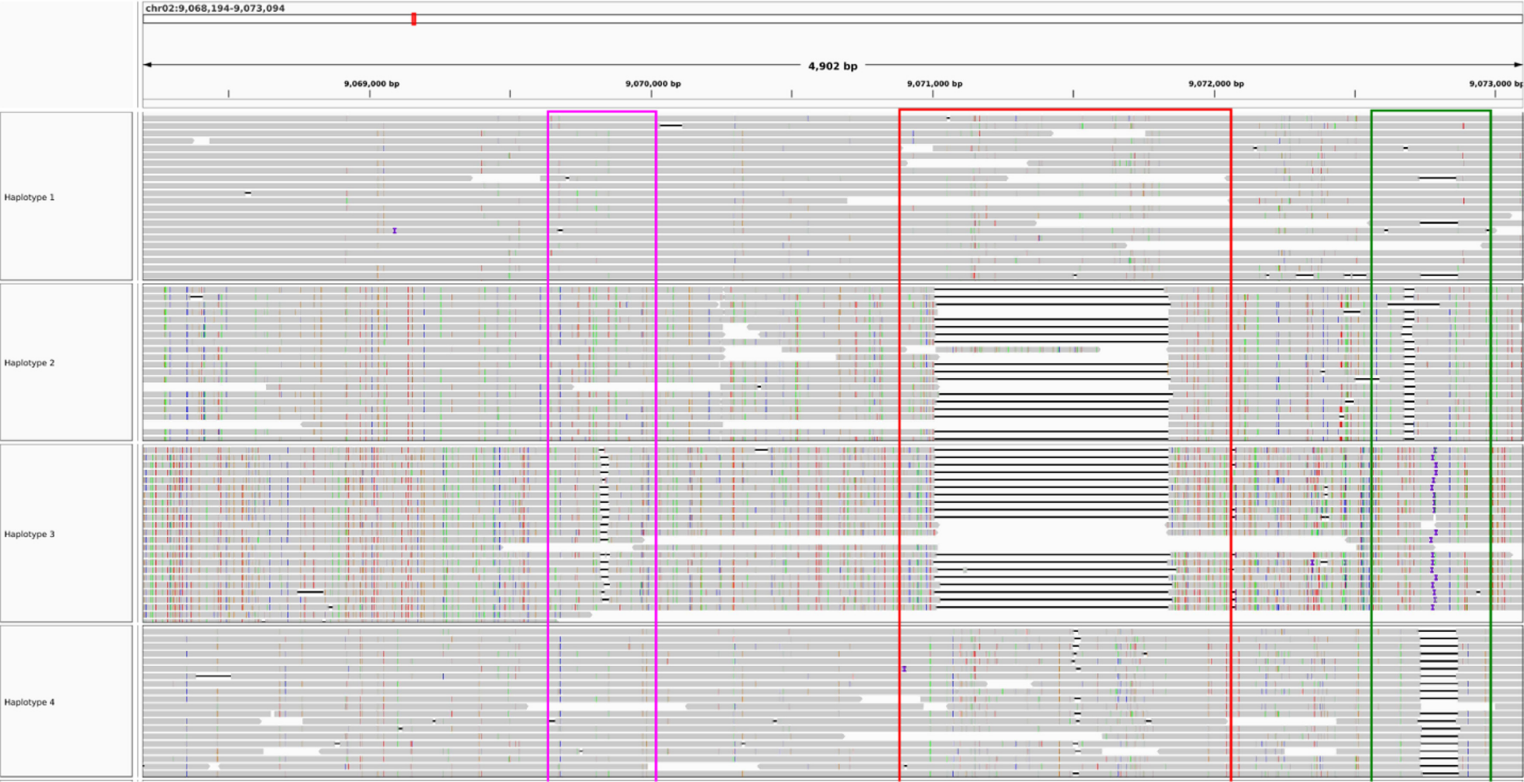
flopp's phasings on a 4902 bp gene. The colored boxes outline structural variations. The clusterings of the structural variations are concordant, implying that the partitioning of reads works reasonably well.

**FIG. 10.**
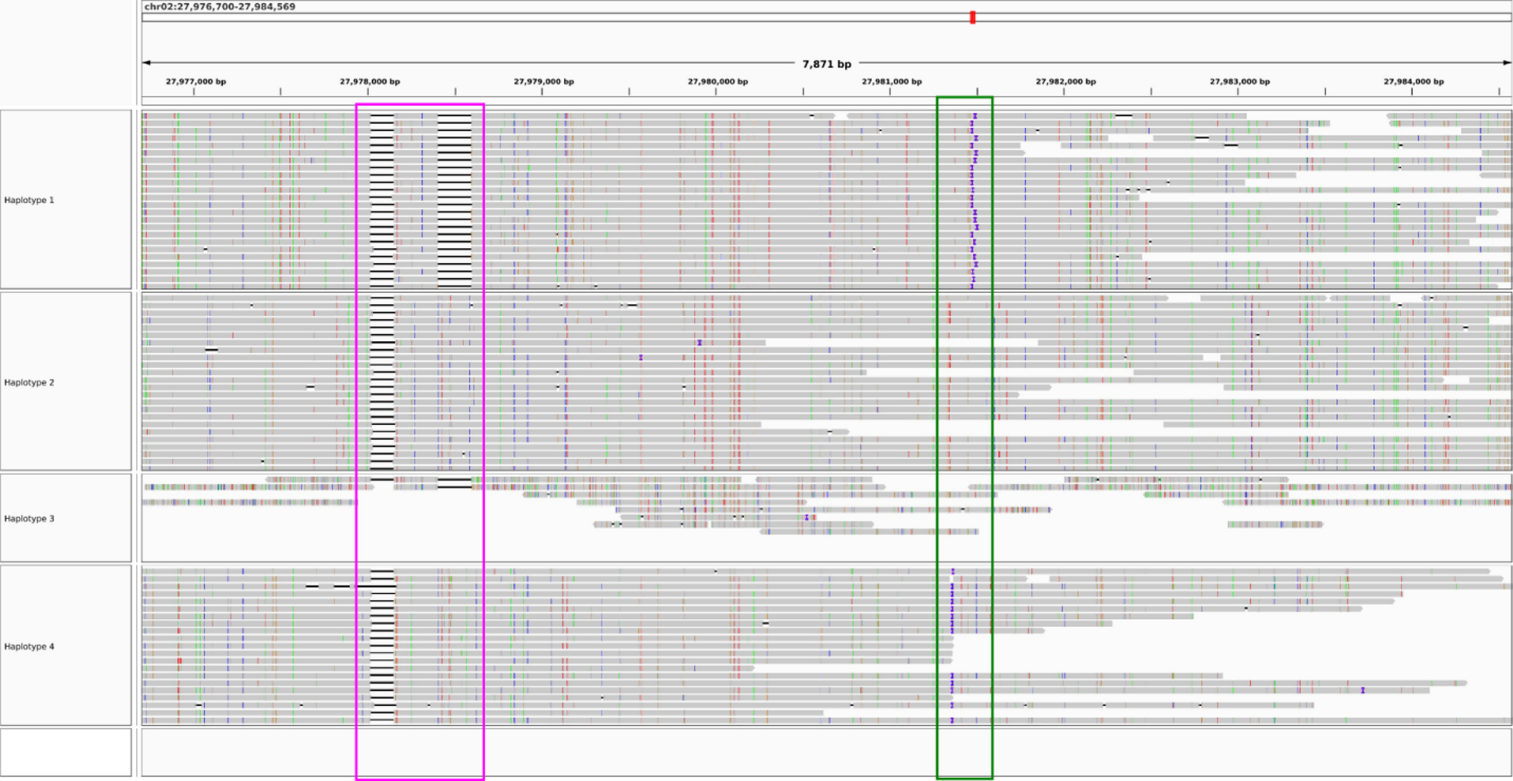
flopp's phasings on a 7871 bp gene. The colored boxes outline structural variations. The phasings for haplotypes 1, 2, and 4 look reasonable, whereas haplotype 3 has very low coverage and it appears that most reads are noisier supplementary alignments. On further inspection, haplotype 2 has twice the coverage of haplotypes 1 and 4, indicating haplotype collapsing and suggesting that haplotype 3 should look similar to haplotype 2.

We ran flopp on real nanopore data sequenced from an autotetraploid potato (*S. tuberosum*) specimen generated from Schrinner et al. (2020). We used the DM v6.1 *S. tuberosum* assembly from Pham et al. (2020) as our reference, and we called SNPs using freebayes (Garrison and Marth, 2012) from short-reads that were also obtained from the same specimen. We aligned all reads with minimap2. We ran flopp on chromosome 2 in the assembly, which was 46 Mb long and had an SNP heterozygosity of 2.0%, that is, 50 bp average distance between SNPs.

flopp only took 1050 seconds to complete on 10 threads. The WHP took 11,492 seconds on the same dataset, and H-PoPG took 8321 seconds. We show two examples of flopp's phasings on two genes in Figures 9 and 10, the first an example of a successful phasing and the second an example of the difficulties of phasing collapsed haplotypes.

In Figure 9, the clustered structural variants between haplotypes show that our phasings are quite good. Note that haplotypes 1 and 4 differed for only 13 out of 180 alleles. Thus, we are able to separate haplotypes that look relatively similar. However, in Figure 10, three of the haplotypes look perfectly phased, whereas haplotype 3 has very low coverage and mostly consists of noisy supplementary alignments. It appears that the UPEM uniformity constraint was not strong enough to deter collapsing in this case because the reads in haplotype 3 were so noisy. We found that haplotype 2 had twice the coverage of haplotypes 1 and 4, suggesting that haplotype 3 should look like a copy of haplotype 2.

Although collapsing is an issue for flopp, we have demonstrated that we can still obtain very sensible local haplotypes, even in the case of collapse. We believe that clever post-processing of flopp's output by looking at coverage can recover correct haplotypes in many instances of collapsing. We envision implementing such a post-processing step in future versions of flopp.

## 4. CONCLUSION

In this article, we presented two new formulations of polyploid phasing, the MSMTP problem and the UPEM model. The SMTP score is a flexible graphical interpretation of haplotype phasing that is related to the MEC score when using a specific weighting on the read-graph, whereas the UPEM score is a superior version of the MEC score when uniformity assumptions are satisfied. Using our probabilistic formulation, we give a new notion of distance between read fragments based on the Kullback-Leibler divergence, which has a rigorous interpretation as a log *p*-value of a one-sided binomial test.

We implemented a fast, local phasing procedure by using these new formulations and showed that our software, flopp, is faster and more accurate on high coverage data while always having extremely accurate local phasings across a range of error profiles, coverages, and ploidies.
